# An Empirical Investigation of Disability-Related Interpersonal Violence Through an Intersectional Research Paradigm: Methodological Considerations and Implications

**DOI:** 10.1177/08862605241270040

**Published:** 2024-09-29

**Authors:** Anastasia Liasidou, Andros Gregoriou

**Affiliations:** 1 St Mary’s University, Twickenham, London, UK; 2 Liverpool John Moores University, UK

**Keywords:** interpersonal violence, intersectionality, disability, gender, race, age, econometric approach

## Abstract

The study uses an econometric approach to disaggregate data on disability-related violence reported in Accident and Emergency departments in London to investigate the extent to which the intersections of gender, ethnicity, and age characteristics of disabled people increase their risk of experiencing interpersonal violence. Our empirical findings suggest that females and older individuals are generally more likely to be interpersonal violence victims. The disability dummy is positive and significant, irrespective of gender or ethnicity. This implies that persons with disabilities are more likely to be victims of interpersonal violence than individuals without disabilities, regardless of gender and ethnicity. The most important discovery concerns the magnitude of the dummy coefficient regarding the disability of individuals. The coefficient is the largest for females of Black origin with disabilities, followed by Asians, with white origin exhibiting the smallest coefficient. This suggests that people with disabilities of Black origin are more likely to experience interpersonal violence than the Asian or white community. The study outcomes provide novel and rigorous empirically validated knowledge of the intersectional vectors of power that impact the risk of experiencing disability-related interpersonal violence while informing the development of intersectionality-based policy approaches to tackling disability-related interpersonal violence.

## Introduction

Disabled people are disproportionately subjected to violent behaviors (Dammeyer & Chapman, 2018; Mueller et al., 2019), and often, they experience a gradual escalation and proliferation of verbal and physical violence. Early subjection to violence, especially in childhood, significantly increases the risk of being subjected to interpersonal violence in adulthood (Fox, 2024) and experiencing the sequelae of debilitating impacts on mental and physical health (R. B. Hughes et al., 2019). Even in cases where experiences of seemingly minor incidences of victimization, such as “invisible injuries” in the form of microaggressions, discrimination, and harassment, are not addressed, they can lead to more severe physical violence and hate crimes (Healy, 2019). Hence, focusing on interpersonal violence may have a broader impact on preventing and addressing other forms of violence experienced by disabled people.

The associations between violence and disability have been well established across diverse contexts and through various methodological approaches documenting that disabled people are more likely to experience interpersonal violence (Harrell, 2016; K. Hughes et al., 2012; R. B. Hughes et al., 2011; Krnjacki et al., 2016; Marge, 2011). When interpreting empirical associations between disability and violence, we should not lose sight that individuals with disabilities do not constitute a homogeneous group (Goodley, 2016; Shifrer & Frederick, 2019). Etherington and Baker (2018) caution against mono-dimensional understandings of human identities that downplay the “unique” and complex ways disabled people might experience interpersonal violence.

Despite the availability of some studies on the intersectional dimensions of violence-exposed disabled people (Casteel et al., 2008; Emerson & Roulstone, 2014; Shaw et al., 2011; Smith, 2007), their interaxial analytical focus is limited to two markers of difference, while their methodological rigor and sample population size need to be strengthened from the perspective of generalization through more comprehensive quantitative studies. For example, Tsai and Venkataramani (2016) provide a very thorough evaluation of regression analysis and interactive effects concerning health disparities. Our analysis goes a step further by accounting for the possible problem of omitted variable bias. For instance, we are modeling violence. There are other factors that we have not considered that may influence violence. Our methodology provides robust estimates given this possibility, which is a very powerful tool for the researcher.

Generally, there is a dearth of studies on gendered differences related to violence-exposed disabled people (Namatovu et al., 2018; Olofsson et al., 2015). This can be attributed to the fact that studies on disabled people who experience violence focus only on women, and even in these cases, data on prevalence is limited (Snæfríðar-og Gunnarsdóttir et al., 2023; Van Der Heijden et al., 2019). Olofsson et al. (2015) document higher rates of violence exposure for both men and women with different disabilities without, however, identifying clear trends in gender-specific adjusted odds ratios of violence exposure, except in the case of increasing age that seems to reduce the odds ratios of violence exposure for both men and women with disabilities. The lack of clear trends is also evidenced in the limited number of existing studies aimed at exploring gender-specific differences in violence exposure for people with disabilities (see Narmatovu et al., 2018).

Significant gender-based differences concerning the likelihood of violence exposure for people with disabilities are reported in three of the five studies reviewed by R. B. Hughes et al. (2011). Disabled women who were recipients of vocational rehabilitation had higher odds of reporting drug-related physical violence than men. In another study, disabled women were at a higher risk of reporting 5-year abuse than men. A third study reported that women were significantly more likely to report exposure to lifetime, adult, and past-year sexual violence. At the same time, their male counterparts were more likely to report exposure to past physical violence. Gender-specific differences were also reported in Krnjacki et al.’s (2016) study, whereby disabled women were more likely to experience sexual and intimate partner violence.

Considering these findings, violence exposure for women with disabilities can erroneously be inferred as being a uniform experience- in some cases linked to specific disability categories, unless there is a parallel consideration of how this experience is shaped and differentiated when these women inhabit multiple marginal social locations that increase their risk of violence exposure (Snæfríðar-og Gunnarsdóttir et al., 2023). Disabled women’s exposure to interpersonal violence seems to be “an intersecting field” constituted by gender-based and disability-based violence that is further compounded when women inhabit additional minoritized social locations such as living in poverty. Krnjacki et al. (2016) highlight, for example, the necessity to examine how disabled women’s exposure to violence differs by their socioeconomic status.

Despite acknowledging the necessity of exploring the intersectional dimensions of the disability and violence couplet, there is a lack of research that examines their complex interactions and impact on disabled women’s subjection to interpersonal violence (Alemu et al., 2023). By implication, it is essential to address the research gap on violence against disabled people at intersections of more than one minoritized status (R. B. Hughes et al., 2011; Mueller et al., 2019). Understanding the nature and magnitude of violence exposure of disabled people necessitates a more fine-grained framing of violence than currently exists, captured through attention to the intersections of “disability” with other markers of difference that enhance a disabled people’s likelihood of being subject to interpersonal violence (Mueller et al., 2019). In this respect, it can be assumed that the more minority identities disabled people have, the more likely it is to experience interpersonal violence. Regarding the co-variable dimensions of risk of violence, it is similarly hypothesized that differences in race, age, and gender can have a singular and collective impact on increasing the risk of being exposed to violence. Violent behaviors against disabled people have a genealogy and are aggravated by stigma-induced ideological presuppositions and structural inequities that must be identified and challenged. As appositely pointed out by Mueller et al. (2019, p. 709): “Ignoring the systematic and intertwined roles of ableism and intersectionality further perpetuates the status quo of oppression.”

Tajima (2021) highlights the critical role of individual researchers and the disciplinary field of interpersonal violence in dismantling systemic inequities and discriminatory regimes that oppress and victimize people with multiple minority identities. Research agendas must explicitly focus on diversity and inclusion to address “the root causes of IPV [interpersonal violence] and the structures that contribute to it” and “expose and show their inner workings” (Tajima, 2021, p. 4981). The aim is to facilitate “practice and policy reforms to achieve just system change” (Tajima, 2021, p. 4955). To this end, “researchers should consider intersectionality and should be transparent where their analyses focus on single identities to the exclusion of other unmeasured identities that may have a bearing on the topic” (Tajima, 2021, p. 4958).

Given the above considerations, this study utilizes an intersectional analytical lens to examine how disability inequality is aggravated by its interaction with other systems of oppression and power imbalances that coalesce to render persons with disabilities more vulnerable to interpersonal violence. In this respect, it is hypothesized that differences in race, age, and gender can have a singular and collective impact on increasing/mitigating disabled peoples’ risk of being exposed to violence.

## Intersectionality, Disability, and IPV

Crenshaw (1991) introduced intersectionality to investigate how power inequities and oppression constitute social identities. She exemplified the intertwined and non-hierarchical gendered, racial, classed, and sexuality dimensions of oppression that were not captured by existing legal frameworks that viewed race and gender as distinct parameters. Intersectionality transcends monolithic perspectives on human identities and seeks to explore how multiple identities coalesce and are subject to cumulative and intersecting forms of oppression and/or privilege.

The overreaching influence of deficit-oriented epistemological and ontological framing of disability is partly the outcome and the reason for the significant absence of disability from intersectional research (Frederick & Shifrer, 2018; Goethals et al., 2015; Nichols & Stahl, 2019). Mueller et al. (2019, p. 709) highlight the necessity to “interrogate the relationship between ableism and disability as an intersectional identity.”This can be achieved by recognizing that “disability is fluid and comes with intersectional social division. . . . It has to do with a narrative in which we must start from the subjective meaning of those who experience this social division” (Rodriguez Martinez, 2022, p. 2). In this respect, the analytical thread explores the constellation of social vulnerabilities, power imbalances, and structural inequities and their mutually reinforcing stigma-induced ideologies that may result in and escalate disabled people’s exposure to interpersonal violence.

In Britain, the notion of intersectionality provided the theoretical lens to problematize and dismantle disability dogmatism linked to “the whiteness and the maleness of the disability rights movement and disability studies” (Evans, 2016, p. 3). Feminist analyses of disability highlighted how Disabilities Studies and the social model of disability ignored the gendered dimension of disability experience and brought to the fore the importance of the personal experience of disability (Barnes & Mercer, 2010; Thomas, 1999, 2013). Even though disability has remained the “master narrative” of the analytical process, this narrative has been enriched and diversified by making salient the “racialized, gendered, and classed” dimensions of disability experience and provided, according to Thomas (1999, p. 120), “counter-narratives” to problematize and challenge reductionist and fixed constructions of disabled people’s identity.

Given the intersectional nature of disability experience, there are no clear-cut disability labels that can fully capture the complex, heterogeneous, and idiosyncratic nature of disability experience. Disabled people cannot be homogenized or categorized according to their “type” of disability and vulnerability to violence, not only because we want to avoid a deficit-oriented framing against which to gage the relationship between disability and violence but also because there is a growing body of research documenting the comorbid nature of disability experiences and the co-existence, for instance, of physical conditions and other comorbid conditions with intellectual disabilities (Kinnear et al., 2018). By implication, the exploration of disability-related violence requires a new perspective that is not delimited to the experiences of violence as the outcome of specific types of disabilities (e.g., physical and mental) but also as the outcome of gendered, racial, and aged identities (Mueller et al., 2019) and their differential impact on the “lived” experience of disability and interpersonal violence. These co-variables, located across intersecting social matrices of oppression and privilege, shape the uniqueness of the disability experience and highlight the need to empirically investigate how disabled people’s intersecting social identities shape and affect their vulnerability to interpersonal violence (Snæfríðar-og Gunnarsdóttir et al., 2023).

Notwithstanding its crucial role in precipitating socially just “political and social change” (Etherington & Baker, 2018, p. 69), intersectionality has been conspicuously absent from disability studies, whose analytical focus was limited to the analogies that can be drawn between the social injustices and police violence experienced by disabled people and racial minority backgrounds. The theoretical analogies failed to acknowledge the cumulative effects of these systems of oppression on the lived experiences of disabled people from racial minorities and underprivileged socioeconomic backgrounds (Frederick & Shifrer, 2018). In this respect, more empirical studies are necessary to provide a comprehensive analysis of the extent to which individuals with disabilities might experience violence not only on the grounds of their “impairments” but also on the grounds of the intersection of their disability status with other minority statuses linked to gender, race, and age.

In parallel, even though K. Hughes et al. (2012, p. 9) conducted a meta-analysis of studies on disability-related violence to suggest that “odds of violence increased in disabled individuals after adjustment for age,” they concomitantly made clear that a knowledge gap exists as “insufficient data were reported in included studies to allow for the calculation of adjusted odds ratios by age or other factors.” Hence, more high-quality population-based studies are needed to enhance understanding of how an intersectional lens can provide a more nuanced and sophisticated analysis of disabled people’s exposure to interpersonal violence.

Even though intersectionality has been widely applied to qualitative studies across different disciplines, including in public health research, there is a lack of quantitative studies that adopt an intersectional perspective (Fehrenbacher & Patel, 2020). Even in these cases, the studies are not explicitly and adequately informed by and framed against an intersectional analytical lens (see Bowleg, 2012; Else-Quest & Hyde, 2016).

Apart from their methodological novelty in developing research designs linked to intersectionality (Hankivsky et al., 2014), quantitative studies enable us to “understand the story of data” (Tabron, 2019, p. 278) on disability-related violence, as well as to provide empirical testing of intersectional theories (Strid et al., 2013, p. 558) and to generate “macro-data” to inform the development of intersectionality-based policies (Etherington & Baker, 2018, p. 69). As suggested by Hankivsky et al. (2014, p. 2), there is a need for “explicit and user-friendly methods” to develop practical applications of intersectionality that policymakers and policy researchers can understand. Contrary to a robust theoretical base against which to examine the reciprocally interacting and interwoven nature of multiple social statuses and power dynamics, there needs to be more methodological choices for policymakers and academics in the empirical investigation of these intersections. Our study will address this need for choices by advancing new interactive methods; the aim is to find a two-way interaction or higher order interactions (three and *n*-way interactions) among the minority status variables under consideration, thereby displaying both analytic sensibilities to the identities of disabled people and empirical robustness. Our methodology thus mitigates some of the limitations of existing intersectional quantitative studies based on their inability to ask questions about intersectionality that are not inherently additive. For instance, Fehrenbacher and Patel (2020, p. 146) highlight how “public health researchers have often oversimplified intersectionality theory by focusing primarily on identity categories as distinct variables rather than interactive processes.” This study will address this crucial knowledge gap on interpersonal violence and disability by accessing a recently developed, extended, high-frequency, novel dataset.

Given the limitations of previous research, along with the fact that our proposed methodology has been rarely used in social science research, our study builds upon an earlier study (Liasidou & Gregoriou, 2021) to transform the understanding of disability and interpersonal violence through the first ever-to our knowledge- the quantitative study of the topic using an intersectional approach and the generalized method of moments (GMM) panel estimator. The study will be innovative by addressing the knowledge gap on how disability inequality is aggravated by its interaction with other systems of oppression and power imbalances that coalesce and render individuals with disabilities more vulnerable to interpersonal violence. In this respect, it is hypothesized that differences in disabled people’s ethnicity, age, and gender can have a singular and collective impact on increasing/mitigating their risk of being exposed to interpersonal violence. By doing this, we can improve policies and initiatives to reduce the high levels of violence affecting disabled people.

## Aims and Objectives of the Study

The overall aim of our study is to use, for the first time, an econometric approach to disaggregate data on disability-related violence reported in Accident and Emergency (A&E) departments in London to investigate the extent to which the intersections of gender, ethnicity, and age characteristics of disabled people increase their risk of experiencing interpersonal violence. The utilization of A&E is justified by the existence of empirical evidence suggesting the “increased use of health care services” by victims of interpersonal violence who experience both physical and mental health effects (Elliott, 2003), while the physicians in A&E departments are “the first professional from whom an abused person seeks help” (Marge, 2011, p. 154).

An intersectional perspective advances anti-essentialist discourses of disability experience that seek to move beyond the polarization of ability/disability dyad and to lay bare the hybrid, fluid, dynamic, and pluralistic nature of disabled identities and their associations with interpersonal violence. This analytical lens can be applied across a continuum of minority statuses and their remote and collective interaction with the “lived” experience of disability across differing intersectional social locations and their associations with interpersonal violence (Mikton & Shakespeare, 2014). The analysis will not be linear; multiple minority statuses do not only act cumulatively and incrementally, but their interaction is complex, idiosyncratic, and unpredictable, and certain minority statuses might mitigate rather than increase the risk of experiencing IPV.

Our study aims to address the following research questions:

1. To what extent do other statuses linked to ethnicity, gender, and age enhance the risk of disability-related violence?
2. Which statuses (gender, ethnicity, and age) are more likely, individually and collectively, to enhance the risk of disability-related violence?
3. What are the policy implications of an intersectional perspective in preventing and dealing with disability-related IPV?

Objectives:

1. To gather data on the impact of differential makers of difference on disability-related violence risk.
2. To test the extent to which disability-related risk is increased/ mitigated due to differential markers of difference.
3. To model an empirically informed and validated intersectionality-based policy analysis and development framework to prevent and address disability-related IPV.

## Methodology: Econometric Specification

To conduct our empirical analysis, the study builds upon previous research (Liasidou & Gregoriou, 2021) to model the relationship between the daily violent injury rate (V) and the disability of individuals by estimating the following equation:

(1) $$V_{it}=\alpha_{i}+b_{t}+\gamma_{1}D_{i}+\gamma_{2}{\mathrm{Age}}_{i}+\gamma_{3}{\mathrm{Gender}}_{i}+\varepsilon_{it}$$

where *i* represents the individuals in our sample and *t* denotes the daily time period; α captures the time-invariant unobserved violent injury rate individual-specific fixed effects (e.g., differences in the injury rate of individuals independent of disability, age, and gender of a person). The $b_{t}$ captures the unobservable individual-invariant time effects (e.g., changes in disability benefits that affect the association between the disability and the rate of violence for all individuals). The empirical relationship between disability and the violent injury rate of individuals is captured by the dummy variable *D_i_*. The dummy takes a value of 1 if the individual has a form of disability and 0 otherwise. Age_*i*_ represents the self-reported age of each individual in our sample. Gender_*i*_ is a dummy variable that is assigned the numerical value of 1 if the individual is female and zero otherwise. Equation (1) is estimated for three different ethnicity groups, namely White, Black, and Asian.^1^

Following Liasidou and Gregoriou (2021), we overcome the potential contemporaneous correlation, endogeneity, and joint determination of the violent injury rate and the disability of an individual by employing the GMM system panel estimator established by Blundell and Bond (1998) on our data.^2^ The standard fixed effects panel estimator does not apply to our econometric analysis because it does not encapsulate the possible contemporaneous correlation across the individuals in our sample. The Three Stage Least Squares panel technique also estimates a system of equations simultaneously and is regarded as an alternative to the GMM system estimator. However, we implement the GMM system, given that it accommodates the possibility of joint determination of an equation system with different instruments for different equations (Schmidt, 1990).^3^

Our GMM system panel estimator allows for joint determination between the independent variable (daily violent injury rate) and all the explanatory variables. For example, our system estimator accounts for the empirical relationship between violence and disability, as well as disability and violence. We are, therefore, computing *n*-way interactions. We acknowledge that we could have estimated different single equation models with various dependent variables. We believe this is not the optimum solution, as the system estimator, which we use, does provide fewer errors in estimation, reflected by the strong diagnostic test results present for all models, in the “Empirical Results” section of the paper.

## Data

In order to conduct our econometric analysis, we extend the dataset used by Liasidou and Gregoriou (2021). This is accomplished by considering the influence of age, gender, and ethnicity on violence. We obtained daily data from 26 major A&E departments in London for each day in 2016.^4^ We establish if the injury is associated with an accident or interpersonal violence from computerized records. Disability (physical or mental) and age are self-reported by the individuals in our sample. Given that prior research has established a relationship between victims of interpersonal violence and disability (Liasidou & Gregoriou, 2021), this motivates us to provide further empirical evidence on the determinants of the victims of interpersonal violence.

The final sample contains a total number of 236,241 observations, with 163,200 from white, 44,880 from Asian, and 28,161 from Black origin.^5^

Our data is obtained from computerized hospital records. One of the major advantages of using hospital data is that it is a high-frequency rich dataset, which allows us to compute robust econometric estimates. It is also a very reliable data source, and patient confidentiality is maintained at all times. The disadvantage is that we do not know how the disability question was phrased, details regarding missing data, and the response rate. Despite these issues with the data, this is a comprehensive, high-frequency, rich dataset that provides a potentially useful contribution to the literature.

## Empirical Results

We report all the empirical findings in Table 1, panels A to C. Panel A looks at the impact of gender, age, and disability on violence for individuals of white origin. Panels B and C examine the association between violence, gender, age, and disability of individuals of Asian and Black origin. The first thing to report is that the fixed and time effects are significant for all estimated models. This implies that the panel estimator is the appropriate econometric specification of our model. We also witness that the analysis does not suffer from any diagnostic problems. For instance, there are no issues of serial correlation, and the empirical estimates are not driven by outliers in the data, as shown by the insignificant Jarque-Bera normality tests. The Sargan tests provide empirical evidence that the instruments for all GMM system estimations are valid. We observe that for all models, gender and age are both positive and significant. This suggests that females and older individuals are more likely to be victims of interpersonal violence. In all panels of Table 1, the disability dummy is positive and significant regardless of gender. This implies that disabled people are more likely to be victims of interpersonal violence. This finding supports Liasidou and Gregoriou (2021), which is expected given that we use a similar dataset. The most interesting discovery concerns the magnitude of the dummy regarding the disability of individuals. The coefficient exhibits the highest magnitude for individuals of a Black origin, followed by Asian, with white origin displaying the smallest coefficient. This suggests that people with disabilities of Black origin are more likely to experience interpersonal violence than the Asian or white community. Our results also reveal that if an individual is female, older, and disabled, they have a greater probability of being the victims of interpersonal violence. We witness that Black individuals who are female, older, and disabled are more likely to be the victims of interpersonal violence than individuals of Asian or White origin. Moreover, our findings suggest that young white males who are not classified as disabled are the least likely to be the victims of interpersonal violence.

**Table 1.** The Empirical Relationship Between Disabled Individuals and Violence for White, Asian, and Black Origins.

Variable	Coefficient
Panel A: White origin	
Constant	18.33 (.00)*
Age	1.26 (.00)*
Gender	3.22 (.00)*
Disability dummy variable	0.44 (.00)*
α_*i*_	(.00)*
$b_{t}$	(.00)*
*SE*	0.61
AR(1)	(.33)
NORM(2)	(.67)
Diff. Sargan	(.53)
Hausman test	42.33
*R*^2^	0.33
Observations	163,200
Panel B: Asian origin	
Constant	21.32 (.00)*
Age	1.82 (.00)*
Gender	3.87 (.00)*
Disability dummy variable	0.61 (.00)*
α_*i*_	(.00)*
$b_{t}$	(.00)*
*SE*	0.66
AR(1)	(.39)
NORM(2)	(.51)
Diff. Sargan	(.41)
Hausman test	49.11
*R*^2^	.21
Observations	44,880
Panel C: Black origin	
Constant	23.98 (.00)*
Age	1.94 (.00)*
Gender	4.11 (.00)*
Disability dummy variable	0.96 (.00)*
α_*i*_	(.00)*
$b_{t}$	(.00)*
*SE*	0.73
AR(1)	(.26)
NORM(2)	(.43)
Diff. Sargan	(.26)
Hausman test	33.23
*R*^2^	.16
Observations	28,161

*Note.* AR(1) is the first order Lagrange Multiplier test for residual serial correlation, undertaken on the residuals of the first difference for the GMM system because of the transformations involved. *SE* represents the standard error of the panel estimator. Sargan tests follow a χ^2^ distribution with *r* degrees of freedom under the null hypothesis of valid instruments. The difference-Sargan test is applicable to the GMM system estimator due to the transformations involved. To establish the validity of the instrument set. NORM(2) is the Jarque-Bera normality test. The Hausman test follows a χ^2^ distribution with 2 degrees of freedom, resulting in a critical value of 5.99, at the 95% confidence level. The endogenous explanatory variables in the panel are GMM instrumented setting, z=1. (.) are *p* values.

* Indicate significant at the 5% level.

We further report that our results are not due to a random error, given that all models do not suffer from any diagnostic problems. If random errors occurred, we would expect some issues with serial correlation and non-normality through fat tails and excess volatility in the data. This further reflects the reliability of our comprehensive large data set and the econometric techniques that we apply.

## Discussion

The study demonstrated how intersectionality can be empirically utilized as a methodological tool to foreground disparities among disabled people who experience interpersonal violence. The study’s empirical findings provide evidence to suggest that in addition to how disability discrimination can explain links between violence and disability (Cea D’Ancona, 2017), these links are further compounded by the cumulative effects of sexism, racism, and ageism. Black disabled women are overall more susceptible to interpersonal violence due to the synergistic effects of the discourses of “whiteness” (Ladson-Billings, 1998) and “patriarchy” (Becker, 1999) that give rise to racial and gendered discrimination that is exacerbated by the mutually reinforcing effects of “ableism” and “ageism.” Even though Olofsson et al.’s (2015) study documents that increasing age seems to reduce the odds ratios of violence exposure for both men and women with disabilities, our findings suggest that older individuals are more likely to be victims of interpersonal violence.

The findings challenge mono-dimensional theorizations of the disability and interpersonal violence couplet that have been routinely framed against calibrations of interpersonal violence victimization based on disability categories (e.g., physical vs mental disabilities) that define and project depoliticized and reductionist understandings of the disability experience. Interpersonal violence on the grounds of disability is enacted in a “discursive crossroad” where differing sources of social disadvantage and discrimination intersect and create the conditions that engender symbolic and actual forms of interpersonal violence experienced by disabled people who are defined as members of more than one minority group. Disability categories that draw a demarcation line between physical, mental, and psychological impairments to explain links between disability and interpersonal violence silence the complexity of disability experience and the differential ways the latter is implicated in hierarchical social structures and relations at the interstices of disability with other minority statuses. Disablism is just one facet of the intertwined vectors of prejudicial structural inequitable conditions of racism, sexism, and ageism that breed interpersonal violence against disabled people and highlight the importance of intersectionality in gauging the extent and how “disability, gender, and race/ethnicity structurally interrelate to establish access to resources (both material and relational) or lead to risks for the groups in question” (Davaki et al., 2013, p. 9).

Regarding policy implications, the study’s outcomes shed light on the role of intersectional discrimination in enhancing disabled people’s vulnerability to violence exposure. This issue is highly relevant to policymakers and service providers, who are expected to improve the lives of disabled people. Intersectionality in policy constitutes a sine qua nonelement in safeguarding disabled people’s human rights by acknowledging that their needs are contingent on the multiplicity of their “social identities” and interactions with systems of privilege/underprivilege (Bowen et al., 2019). As Mueller et al. (2019, p. 721) pointed out, “. . . aggregating results into a single binary [dis/abled] . . . may miss indicators of risk factors and opportunities for targeted interventions.” An intersectionality-based approach involves the parallel exploration of distinct and interactive effects on violence prevention work (Davaki et al., 2013). Applying this approach to disability-related violence prevention work brings to the fore the mutually reinforcing interactions among differential inequalities and discriminatory regimes dissimilar to their distinct instantiations and ramifications (what each source of disadvantage would have produced singlehandedly).

## Conclusions

Our research used empirical evidence to theorize and concretize implications for violence prevention work while rationalizing the necessity to embed an intersectional perspective into public policy to prevent and tackle disability-related interpersonal violence. Tackling disability-related violence through various policies and interventions necessitates addressing the pyramid of violence that reflects a continuum of prejudicial attitudes, discriminatory regimes, microaggressions, and verbal abuse that are mutually reinforcing and escalate into physical violence. This signifies the importance of our study not only due to the prevalence of physical injury in interpersonal but also due to the anticipated contribution of the study to reducing other forms of violence.

Our quantitative intersectional inquiry provides fertile ground to develop new and credible empirically validated knowledge of the intersectoral vectors of power that are played out and impact the risk of experiencing disability-related interpersonal violence. The latter is instigated and perpetuated by systemic inequalities and presumed deviations from notions of “normalcy” that lie across the intersections of disability with other markers of difference. These intersections have varied degrees of causal effects on the experience of disability-related violence and can inform the formulation of more effective and equitable social and public health policies (Bowleg, 2012).

An intersectional approach to policy constitution and dissemination can address the genealogy of disability-related violence (Goodley & Runswick-Cole, 2011), by exposing the reciprocally related systems of oppression that converge to enhance vulnerability to interpersonal violence. This necessitates developing “joined up” policies to challenge the simultaneous discrimination and poly-victimization experienced by disabled individuals as members of more than one minority group (Brassard et al., 2015). Therefore, social and health-related policy developments and anti-discrimination legislation should be informed by an intersectional paradigm to address differential community needs and identify the genealogy of social problems and discriminatory regimes (Bowen et al., 2019) linked to interpersonal violence.

Mitigating the factors and consequences of disability-related interpersonal violence also necessitates developing professional awareness of the “impacts of intersecting marginalized identities” to address “both immediate individual barriers and longstanding systemic issues that perpetuate the abuse of people with disabilities” (Lund, 2020, pp. 202–203). Davaki et al. (2013, p. 16) are explicit about the significance of research not only “as a means of evidence-based policy making but also as a way to raise awareness.” Hence, in addition to developing intersectionality-based policy approaches to tackling interpersonal violence, professionals and policymakers should develop an understanding of intersections of various dimensions of trauma linked to interpersonal violence with structural inequities, as well as minority statuses linked to gender and race (Bowen et al., 2019) in shaping the “lived” experience of interpersonal violence exposure. This understanding also involves developing a critical awareness of how ableism in society and within themselves interacts with racism, sexism, and ageism and contributes to the dehumanization and subjugation of “disabled lives” (Fox, 2024; Lund, 2020).

This eco-systemic approach promotes “the person-in-environment-perspective” (Levenson, 2017, p. 105) on understanding and dealing with the intertwined and reciprocally related “ecologies” that shape disabled people’s experiences of interpersonal violence while also considering the interactions of gender and race and their intersections in examining the disability, trauma and interpersonal violence nexus. An intersectional paradigm affords policymakers and professionals conceptual and pragmatic tools to address the root causes of violent behaviors toward disabled people living at these critical, albeit largely unexplored, intersections.

Our methodology provides reliable estimations in the presence of omitted variables. This is a very powerful tool for researchers to have at their fingertips. When they are modeling violence, diseases, etc. there are many factors that are not considered. This is due to lack of time and data availability. We encourage future researchers to implement this methodology when they conduct their empirical investigations. In order to accomplish this, researchers can use the STATA command xtxtdpdsys (https://www.stata.com/manuals/xtxtdpdsys.pdf).

## Limitations of the Study

Even though our methodological approach demonstrates analytic sensibility in elucidating how a disabled person’s social locations across some minority statuses influence their odds of experiencing interpersonal violence, a mixed methods approach could have provided more nuanced insights into the context-specific dimensions and subtleties of this experience (see Fehrenbacher & Patel, 2020). A multi-method approach would have facilitated a better understanding of the complex interplay of intersectional identities and system-wide realities that influence the extent to which and how multiply marginalized disabled people experience interpersonal violence (Mueller et al., 2019, p. 708).

Another limitation of our study is that we do not know how the disability question was phrased, the details of missing data, or the response rate. Despite these limitations, this comprehensive, high-frequency, and rich dataset contributes to the literature. However, it also needs to be noted that even when the disability question phrasing is known, a recurrent problem found in similar studies is, according to R. B. Hughes et al. (2011), their failure to provide a uniform, unequivocal, and consistent definition of disability across studies. The problem of “terminological messiness” around disability is attributed to the complex, contested, and highly politicized nature of disability, which is understood, shaped, and defined differently across diverse sociopolitical contexts (Alemu et al., 2023). The contextually mediated character of disability is compounded in our research by the fact that disability is a self-reported social identity. A further limitation relates to the data that does not include disabled people from specific racial minorities (e.g., Middle Eastern, Hispanic, Indian, Chinese, and multiracial people) alongside other intersecting markers of difference (e.g., social class, culture, ethnicity, and sexual orientation) that can enhance their susceptibility to experience interpersonal violence. If the data is available in the future, this would be an interesting avenue for further research.

## Notes

1. Liasidou and Gregoriou (2021) also use the price of alcohol as an additional control variable in their research. We do not include this in our empirical analysis as it is not the purpose of our study.

2. The Hausman (1978) test establishes that the explanatory variables in Equation (1) are endogenous. These results are not reported but are available from the authors upon request.

3. Our econometric model has a one lag structure. For more information on the GMM system Panel estimator readers are referred to Liasidou and Gregoriou (2021). Liasidou and Gregoriou (2021) also empirically examine data in 2016.

4. A list of the 26 A&E departments that took part in our study is available from the authors upon request.

5. Liasidou and Gregoriou (2021) had a total of 408,000 observations in their sample. Our dataset is substantially reduced as we only consider individuals who have given information concerning their age and are of white, Asian, or Black origin.
